# Harnessing Phase Separation in the Extracellular Matrix: From Mechanisms to Therapeutic Opportunities

**DOI:** 10.1155/bmri/1522109

**Published:** 2026-01-21

**Authors:** Runhan Guo, Jiayi Li, Xuenan Wang, Huabao Xiong, Fei Wu, Tao Zhong

**Affiliations:** ^1^ Institute of Immunology and Molecular Medicine, Key Laboratory of Cell and Biomedical Technology of Shandong Province, School of Basic Medical Sciences, Jining Medical University, Jining, China, jnmc.edu.cn; ^2^ Affiliated Hospital of the Jining Medical University, Jining, Shandong, China; ^3^ Department of Urology, Shandong Provincial Hospital Affiliated to Shandong First Medical University, Jinan, Shandong, China, sph.com.cn

**Keywords:** extracellular matrix, phase separation, therapeutic strategies

## Abstract

The extracellular matrix (ECM) constitutes a sophisticated network that is paramount in preserving tissue integrity and modulating cellular behaviors. Recent revelations regarding phase separation provide a groundbreaking perspective on the ECM, elucidating how this phenomenon affects the spatial organization and functionality of its components. This review delves into the implications of phase separation in ECM‐related pathologies and underscores its prospective role as a therapeutic target. By adeptly manipulating phase separation, pioneering therapeutic strategies can be devised to restore the equilibrium of the ECM, employing biomimetic materials and cutting‐edge drug delivery systems to advance tissue repair and regeneration. Such innovative approaches hold the promise of enhancing treatment outcomes by directly addressing the molecular mechanisms that underpin ECM dysfunction. Ongoing research into phase separation is imperative for translating these insights into clinical applications, potentially transforming therapies for intricate ECM disorders and offering renewed hope for improved patient care.

## 1. Introduction

The extracellular matrix (ECM) represents an intricate tapestry of proteins, carbohydrates, and polysaccharides that resides in the interstitial spaces between cells, providing vital structural and biochemical support necessary for preserving tissue integrity, facilitating signal transduction, regulating cellular behavior, and promoting tissue repair and regeneration [1]. Through its interactions with cell surface receptors, the ECM exerts a profound influence on cell proliferation, differentiation, and migration, ultimately impacting a myriad of physiological processes [2–5]. Aberrations within the ECM have been implicated in a range of diseases, including tissue fibrosis, tumor metastasis, and neurodegenerative disorders [6–8]. These conditions frequently entail modifications in ECM composition and architecture, which disrupt the cellular milieu and propel pathological mechanisms.

Emerging research indicates that certain ECM proteins may exploit the phenomenon of phase separation to modulate their structural and functional attributes [9]. This revelation offers a novel lens through which to examine the ECM′s capacity to orchestrate complex regulatory functions beyond the confines of individual cells, showcasing a remarkable diversity in both health and disease. Nevertheless, the precise role of phase separation in shaping the intricacy and variability of ECM regulatory functions across healthy and diseased states remains to be elucidated. This review is aimed at investigating the mechanisms by which ECM molecules engage in phase separation in the context of related diseases, with a particular focus on strategies to regulate this process as a therapeutic avenue for conditions stemming from ECM imbalances. Ongoing research is anticipated to illuminate these multifaceted interactions, fostering the development of innovative therapeutic modalities and ultimately enhancing patient outcomes.

## 2. ECM Composition and Function

**Figure 1:**
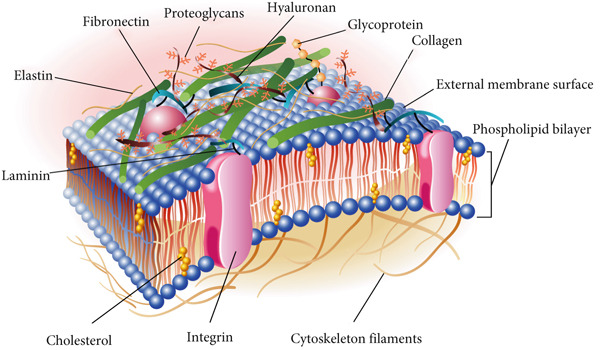
Diagrammatic representation of the extracellular matrix and its key constituents. The ECM′s composition, which differs across tissues, predominantly consists of fibrous proteins such as collagen, elastin, fibronectin, and laminin, alongside polysaccharides. These elements are secreted by cells and subsequently organized into a structured network intimately associated with the originating cell′s surface.

ECM is a dynamic and elaborate network of macromolecules, encompassing proteins, glycoproteins, and polysaccharides that permeate the intercellular spaces within tissues. It serves as a crucial structural framework, providing the mechanical support indispensable for preserving tissue integrity and shape (Figure 1). Beyond its architectural function, the ECM exerts a regulatory influence on cellular behavior through its biochemical and biomechanical properties, affecting cell proliferation, differentiation, migration, and survival via interactions with cell surface receptors such as integrins [10]. These interactions are critical for maintaining tissue homeostasis and coordinating cellular responses to the microenvironment [11, 12].

The ECM is pivotal in tissue repair and regeneration [13, 14]. For example, elastin and collagen fibers undergo dynamic remodeling to facilitate cell recruitment and support wound healing [15]. These signaling cues activate fibroblasts and endothelial cells, which are essential for new tissue formation and vascular network restoration [16, 17]. By adeptly orchestrating these processes, the ECM ensures the efficient resolution of damage while preserving the structural and functional integrity of tissues.

The ECM is not static; it undergoes continuous remodeling to adapt to physiological demands and external stimuli, a balance that is critical for organ function and overall physiological stability [18]. Enzymes such as matrix metalloproteinases play a pivotal role in regulating its composition by degrading aged or damaged components, while cells synthesize new matrix molecules to replenish them [19]. This dynamic remodeling process ensures that the ECM retains its functionality under varying conditions. However, dysregulation of these processes can lead to ECM‐related diseases. Abnormal matrix remodeling disrupts integrin‐mediated signaling, compromising cellular functions and repair mechanisms [20–22]. In fibrosis, excessive accumulation of ECM proteins, particularly collagen, compromises organ structure and function [23–26]. In cancer, the altered matrix creates a permissive microenvironment conducive to tumor progression and metastasis [27–29]. In osteoarthritis, aberrant ECM remodeling instigates chronic inflammation and tissue destruction [30]. In addition, the ECM also plays a critical role in the central nervous system (CNS), where it supports neuronal survival, axonal guidance, and synaptic plasticity [31, 32]. The dysregulation of ECM composition and remodeling in the CNS has been implicated in neurodegenerative diseases such as Alzheimer′s disease (AD) and Parkinson′s disease, where aberrant phase separation of ECM components may exacerbate pathological protein aggregation and inflammation [33].

The profound ramifications of ECM dysregulation underscore its pivotal role in preserving cellular and tissue homeostasis. Whether through the disruption of signaling pathways, the compromise of structural integrity, or the impairment of cellular functions, aberrations in matrix regulation significantly contribute to the progression of various pathological states. A comprehensive understanding of the mechanisms by which matrix remodeling influences disease pathology establishes a crucial foundation for the development of targeted therapeutic strategies aimed at reinstating normal matrix function and alleviating the impact of disease.

## 3. Phase Separation and Its Regulation Factors

Phase separation in biological systems is intricately orchestrated by biophysical principles that are fundamentally rooted in molecular interactions and environmental conditions. Cells harness the phenomenon of phase separation to compartmentalize biomolecules into distinct, membraneless structures. This remarkable occurrence arises from specific interactions among proteins, nucleic acids, and other macromolecules, primarily mediated by weak, multivalent forces. Such interactions enable the transition of molecules from a homogeneous distribution to condensed, droplet‐like states, resulting in the formation of dynamic and reversible assemblies. A key determinant in this process is the delicate equilibrium between attractive and repulsive forces, which governs molecular aggregation while simultaneously preserving the fluidity and functionality of the system [34].

The role of intrinsically disordered regions (IDRs) is of paramount significance. Proteins characterized by unstructured regions facilitate multivalent interactions through repeated binding motifs or low‐complexity domains, thereby fostering the formation of dense molecular networks (Figure 2a). These interactions encourage the clustering of components into specific locales, culminating in the generation of biomolecular condensates [35]. Charged residues within polypeptides and nucleic acids establish electrostatic complementarity, while hydrophobic domains contribute to the energetic stability of these condensates. Collectively, these intermolecular forces propel the assembly of molecules into phase‐separated states. Proteins possessing IDRs or low‐complexity domains frequently function as molecular scaffolds, offering multivalent interaction sites that facilitate the assembly of biomolecular condensates. These dynamic structures are upheld through weak, transient interactions, ensuring their reversible and tunable characteristics. Furthermore, posttranslational modifications such as phosphorylation, ubiquitination, and acetylation fine‐tune interaction affinities, either enhancing or inhibiting phase separation and thereby providing precise regulatory control over cellular processes [36]. Notably, posttranslational modifications regulate ECM remodeling by modulating the phase separation of key ECM proteins or their regulators. For example, serine phosphorylation of ECM structural protein fibronectin (FN1) weakens multivalent interactions between its IDRs, inhibiting liquid–liquid phase separation (LLPS) to prevent abnormal aggregation and maintain ordered ECM fibril assembly, avoiding fibrosis [37]. Conversely, O‐GlcNAcylation of ECM‐associated YTHDF1 boosts its phase separation; YTHDF1 condensates stabilize collagen I‐encoding mRNAs to promote ECM deposition, and its dysregulation links to hepatic fibrosis [38]. Additionally, threonine 114 phosphorylation of cardiac ECM regulator VGLL3 enhances its binding to transcriptional cofactors and phase separation via its low‐complexity domain, driving collagen gene expression and cardiac fibrosis, while PP2A‐mediated dephosphorylation disrupts its condensates to reverse collagen overproduction [39]. These show PTMs act as “molecular switches” to fine‐tune ECM‐related protein phase separation, governing ECM synthesis, assembly, and degradation.

Figure 2Regulation of phase separation and some contributing factors. (a) Proteins containing intrinsically disordered regions (IDRs) (either at the N‐terminus or C‐terminus) drive the formation of liquid‐like phase‐separated condensates: IDRs mediate multivalent transient interactions (e.g., hydrophobic and electrostatic interactions) to form dense molecular networks, which is the core basis for condensate assembly [35]. For example, the IDRs of elastin enable its liquid structure and further support phase separation [47]. (b) Environmental parameters dynamically modulate phase separation behavior: Temperature alters the kinetic energy of molecules and the strength of intermolecular interactions, thereby affecting the tendency of compartmentalization [40]; pH changes the protonation state of amino acid residues, which may enhance or disrupt electrostatic interactions [41]. Ionic strength mediates charge screening to regulate the attraction between oppositely charged molecules [42]. (c) Phase separation exhibits a highly cooperative, concentration‐dependent threshold, defined by a narrow range of physiological concentrations (indicated by blue dashed bar lines). In Square 1, protein concentration is insufficient for phase separation. In Square 2, protein concentration reaches a critical level, approaching the phase separation threshold. In Square 3, proteins surpass this threshold, resulting in condensate formation [44].

**Figure:**
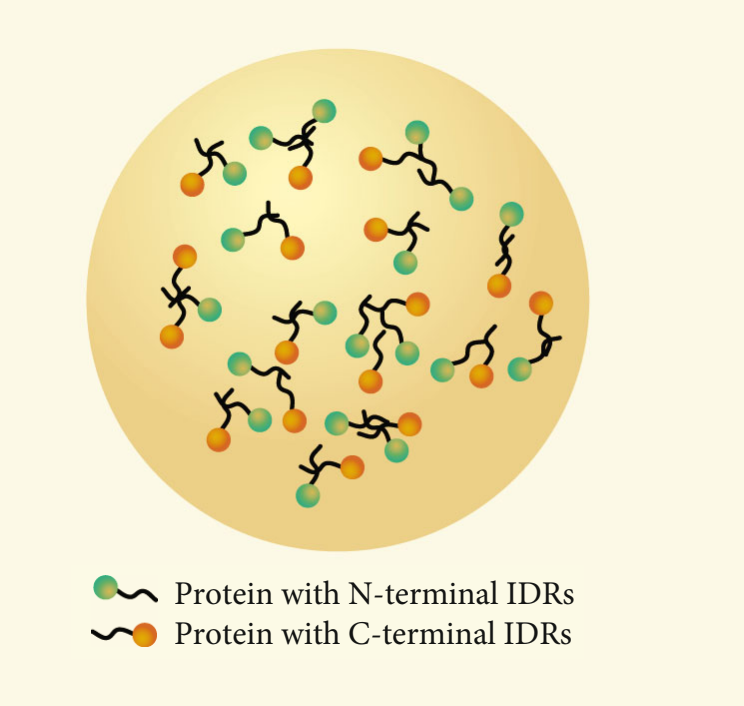
(a)

**Figure:**
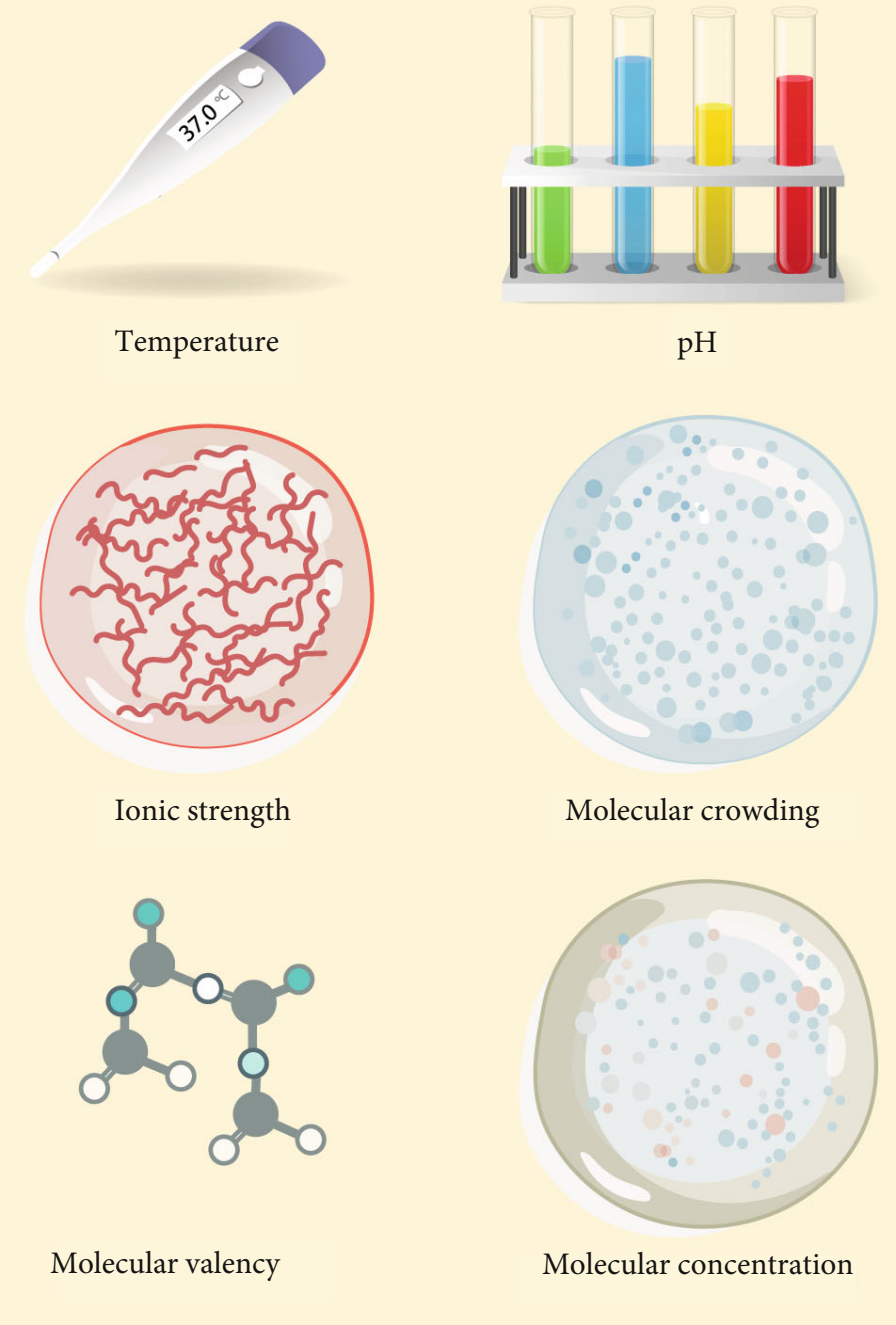
(b)

**Figure:**
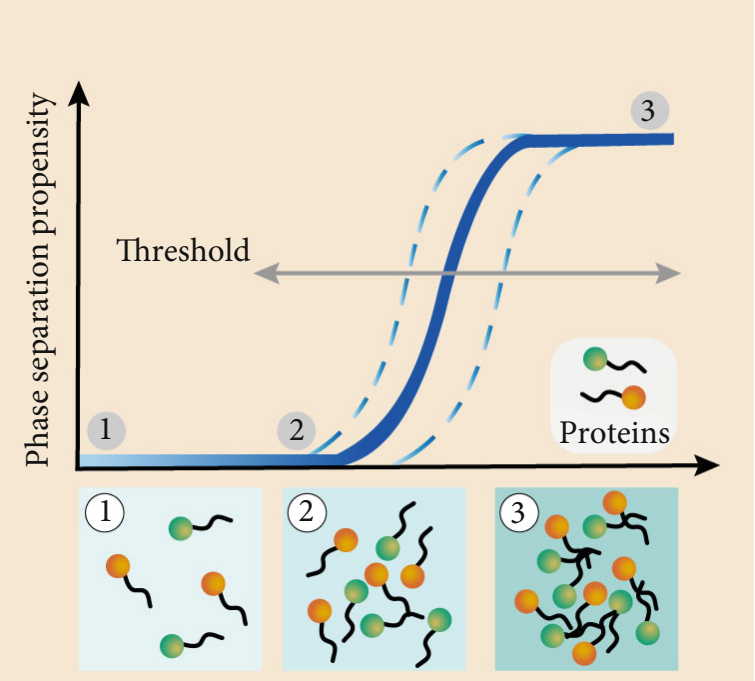
(c)

Environmental factors such as temperature, pH, ionic strength, and molecular crowding exert a profound influence on phase separation behavior. Shifts in temperature modulate molecular kinetics and the strength of interactions, thereby affecting the propensity for compartmentalization [40]. Variations in pH can alter the protonation states of amino acid residues, which may either disrupt or enhance electrostatic interactions [41]. Ionic strength plays a critical role in charge screening, consequently modifying the attractions between oppositely charged molecules [42]. Similarly, the densely packed intracellular milieu, characterized by elevated concentrations of macromolecules, facilitates phase separation by diminishing the effective volume available for molecular diffusion [43] (Figure 2b). Collectively, these factors delineate the concentration thresholds necessary for phase separation and influence the physical properties of condensates. Fluctuations in these environmental conditions ensure that phase separation remains acutely responsive to the dynamic states of the cell.

The principles of phase separation are intricately sensitive to molecular valency and concentration (Figure 2b). Proteins possessing multiple interaction sites facilitate the formation of extensive networks, thereby amplifying the likelihood of condensation [44]. For phase‐separating components to demix into distinct phases, critical concentration thresholds must be exceeded (Figure 2c). Moreover, the presence of specific cofactors or auxiliary elements can modulate the dynamics and composition of phase‐separated systems, ensuring their responsiveness to the ever‐changing demands of cellular processes [45, 46].

The intrinsic dynamism of phase separation is vividly illustrated in condensates that exhibit liquid‐like properties, such as fusion and adaptability to external forces. This dynamic behavior arises from transient and reversible intermolecular interactions, enabling phase‐separated structures to function as pivotal hubs for biochemical reactions, molecular sequestration, and signal transduction. The rapid formation and dissolution of these structures are essential for processes such as transcription and the cellular stress response [48–50]. However, dysregulation of these interactions or imbalances within the environment can precipitate pathological states, contributing to conditions such as neurodegeneration, cancer, and diseases related to the ECM [9, 51, 52]. A deeper understanding of the molecular principles that govern the regulation of phase separation offers critical insights into cellular organization and underscores potential avenues for therapeutic intervention.

## 4. Phase Separation Regulates ECM Disorders

### 4.1. The Role of Phase Separation in ECM Dynamics

Phase separation plays a pivotal role in orchestrating the molecular interactions that regulate the structural organization and functional responsiveness of the ECM. Within the extracellular environment, phase separation facilitates the compartmentalization of matrix components, resulting in the formation of distinct biomolecular assemblies that govern the spatial and temporal regulation of matrix behavior. The interconnectedness of these molecular condensates ensures that matrix functions are meticulously aligned with essential cellular processes, such as adhesion, migration, and differentiation [53].

Phase separation empowers the ECM to regulate the assembly of fibrillar networks and the spatial distribution of matrix proteins [54, 55]. Proteins such as collagen and elastin undergo the formation of structured assemblies via phase‐separated intermediates, thereby enhancing their hierarchical organization [56, 57]. These dynamic interactions ensure the adaptability of ECM mechanical properties, such as elasticity and tensile strength, which are critical for tissue integrity and functionality. The reversibility of phase‐separated states enables the ECM to respond rapidly to environmental changes, thereby maintaining matrix functionality and structural integrity under dynamic conditions.

The molecular foundation of phase separation in the dynamics of the ECM highlights the significance of multivalent interactions and IDRs in driving this phenomenon. For example, IDRs in elastin drive LLPS through transient, multivalent binding interactions [47]. These interactions are further refined by posttranslational modifications, including phosphorylation, glycosylation, and cross‐linking, which modulate the physicochemical properties of matrix condensates [37, 38, 58] and enable the ECM to attain a balance between stability and flexibility, which is crucial for its multifaceted biological functions.

**Figure 3:**
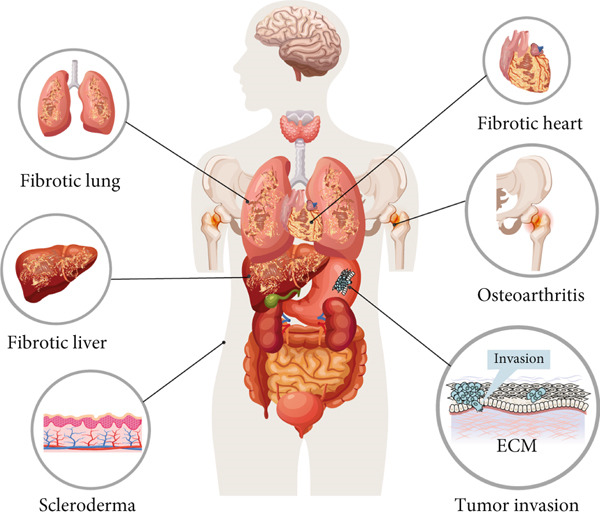
Phase separation plays a crucial role in orchestrating the dynamics, composition, and functionality of the ECM, carrying significant implications for various pathological conditions. Vestigial‐like family member 3 employs liquid–liquid phase separation through its low complexity domain to enhance collagen synthesis, thereby contributing to cardiac fibrosis [39]. Dysregulated phase separation of fibronectin is associated with the excessive deposition of ECM components, a hallmark of diseases such as pulmonary fibrosis, hepatic fibrosis, and scleroderma [59]. Moreover, aberrant phase separation of ECM proteins leads to the pathological accumulation and mineralization of the matrix, a process that undermines joint stability and function, as observed in osteoarthritis [69]. The upregulation of ECM protein matrilin‐3, undergoing phase separation mediated by its low complexity and coiled‐coil domains, significantly promotes gastric cancer cell invasion, correlating with poorer prognosis in affected patients [59].

In pathological conditions, disruptions in phase separation mechanisms can lead to dysregulated matrix dynamics, contributing to the progression of various diseases. Aberrant phase behavior may result in altered matrix composition, excessive stiffness, or impaired degradation, which are hallmarks of diseases such as fibrosis and scleroderma [39, 59] (Figure 3). Consequently, modulating phase separation presents a promising therapeutic strategy to restore normal matrix function and impede disease progression.

### 4.2. Impact of Phase Separation on Matrix Composition and Functionality

Phase‐separating proteins form dense clusters via multivalent domains, recruiting ECM‐critical enzymatic modulators, growth factors, and signaling molecules to drive localized reactions (cross‐linking, proteolysis, and posttranslational modifications) that maintain matrix homeostasis [60, 61]. Disruptions in phase separation cause biochemical processes and matrix‐related pathologies.

Phase‐separated microenvironments may create protective niches that promote selective turnover of damaged components [62]. Furthermore, the inherently dynamic nature of these condensates permits swift reorganization in response to extracellular stimuli, thereby ensuring the adaptability of the matrix under both physiological and pathological conditions [63, 64].

The intricate interplay between phase separation and the functionality of the ECM is prominently manifested in a variety of biological processes, including tissue development, wound healing, and the progression of diseases [65, 66]. For example, the assembly of fibronectin into fibrils is driven by phase separation [67], which facilitates its integration into the matrix and subsequent interactions with integrins and other proteins [68]. Likewise, collagen fibrillogenesis depends on the phase separation state to regulate the nucleation and alignment of collagen molecules [56]. Disruptions in phase separation can perturb these critical processes, contributing to matrix disorders such as fibrosis, osteoarthritis, and tumor invasion [39, 69, 70] (Figure 3).

The regulatory role of phase separation in ECM functionality is particularly prominent in the CNS, where it orchestrates the assembly and turnover of CNS‐specific ECM components critical for neurodegenerative pathogenesis. Tau, a microtubule‐associated protein linked to AD and tauopathies, undergoes LLPS via its IDRs; this phase transition is further modulated by CNS ECM proteins (e.g., fibronectin) [8, 51]. Rare genetic variations in FN1 have been shown to protect against APOE*ε*4‐associated AD, possibly by interfering with Tau′s abnormal phase separation and reducing its aggregation on the ECM [8]. Similarly, aberrant phase separation of ECM proteins (e.g., laminin) promotes the deposition of amyloid‐beta (A*β*) peptides on the CNS ECM, forming neurofibrillary tangles and senile plaques—the hallmark lesions of AD [71]. These findings highlight that phase separation–mediated CNS ECM remodeling is a key unifying mechanism in neurodegenerative diseases.

Gaining insight into how phase separation affects the composition and functionality of the ECM illuminates its critical role in both tissue physiology and pathology. Such understanding presents promising avenues for developing therapeutic strategies that specifically target phase separation processes with the aim of restoring or modulating matrix homeostasis. By harnessing the principles of phase separation, innovative approaches can be devised to regulate protein organization, interactions, and biochemical properties, thereby paving the way for groundbreaking treatments for diseases associated with the ECM.

## 5. Targeting Phase Separation for Novel Treatments in ECM Diseases

### 5.1. Targeting Phase Separation Mechanisms in ECM Remodeling Disorders

Recent advancements in our understanding of phase separation have unveiled valuable insights into its pivotal role in regulating protein assemblies, nucleic acid interactions, and enzymatic activities pertinent to the turnover of the ECM [64, 72, 73]. Aberrant phase behavior has been associated with the aggregation of crucial structural or signaling components, thereby disrupting the normal architecture of the ECM [55, 65]. Therapeutic interventions that target these interactions could mitigate the formation of dysfunctional materials, thereby preserving structural integrity. While the therapeutic landscape is still evolving, several pioneering preclinical studies have begun to validate the feasibility of targeting phase separation in ECM disorders. For instance, in cardiac fibrosis, the mechanosensitive protein VGLL3 drives aberrant collagen production through LLPS. Critically, disrupting VGLL3 condensation has been shown to attenuate collagen deposition and improve cardiac function in mouse models, providing direct proof‐of‐concept that targeting a specific phase‐separating effector can alleviate fibrotic pathology [39]. Similarly, in gastric cancer, the ECM protein matrilin‐3 (MATN3) forms proinvasive condensates via its low‐complexity domain. Notably, genetic or pharmacological disruption of MATN3 phase separation significantly impedes cancer cell invasion in vitro and in vivo, directly linking the phase separation of an ECM component to disease progression and nominating it as a druggable target [59]. Beyond these direct interventions, fundamental research on elastin assembly, a process governed by phase separation, suggests that modulating the physicochemical environment presents another viable strategy to correct aberrant ECM organization [65].

Targeting phase separation also facilitates the precise regulation of cellular signaling pathways associated with ECM remodeling. Liquid‐like compartments generated through phase separation orchestrate the spatial and temporal organization of signaling cascades, thereby influencing the activity of critical molecules such as growth factors, cytokines, and matrix metalloproteinases. Dysregulation of these compartments can intensify pathological signals, exacerbating matrix‐related disorders [59]. Therapeutics crafted to stabilize or disrupt specific phase‐separated assemblies have the potential to modulate these pathways, thereby restoring normal signaling dynamics. This innovative approach could prove particularly advantageous in conditions such as cardiac fibrosis, where aberrant signaling drives maladaptive remodeling and contributes to tissue stiffness [39].

Furthermore, the mechanisms underlying phase separation offer a robust framework for the development of biomimetic materials and drug delivery systems specifically designed to target matrix remodeling [74]. By harnessing the principles of liquid–liquid demixing, therapeutic constructs can be crafted to emulate natural compartments, thereby enhancing the stability, localization, and activity of therapeutic agents [75, 76]. Such materials could be engineered to release drugs selectively within pathological regions, thereby improving efficacy while minimizing off‐target effects [77]. This strategy resonates with the dynamic nature of ECM disorders, which frequently involve localized disruptions in tissue structure and function.

While the therapeutic potential of targeting phase separation is vast, significant challenges persist in fully translating these concepts into clinical applications. Key hurdles include the development of highly specific inhibitors capable of disrupting pathological phase‐separated condensates without interfering with essential physiological processes. Furthermore, the in vivo delivery, stability, and pharmacokinetics of molecules targeting protein–protein interactions within condensates require rigorous optimization to ensure they reach target tissues at effective concentrations without causing off‐target effects. The context‐dependent nature of phase separation, which is sensitive to local concentration, pH, and posttranslational modifications, adds another layer of complexity for reliable therapeutic targeting. Therefore, despite compelling preclinical validations, a substantial amount of research is still imperative to translate these pioneering findings into viable clinical therapies.

### 5.2. Innovative Therapeutic Approaches Leveraging Phase Separation in Tissue Repair

Biomaterials inspired by phase separation have been ingeniously engineered to modulate matrix assembly and foster functional recovery. These materials leverage the capability to form phase‐separated compartments that sequester bioactive molecules, facilitating their precise release in areas of injury. Their tunable properties enable the dynamic regulation of ECM polymerization, thereby enhancing the structural organization of tissues during regeneration [78]. Moreover, phase‐separated hydrogels can effectively mimic the viscoelasticity of the native environment, providing essential mechanical support that promotes cellular adhesion, proliferation, and differentiation [79]. Such materials create an optimized repair niche that aligns harmoniously with the physiological needs of damaged tissues [80].

Targeting cellular interactions through phase separation presents another promising avenue for therapeutic innovation. Cellular behavior within tissues is intricately governed by complex interactions with the surrounding matrix, mediated by biochemical gradients and mechanical feedback [81, 82]. By harnessing the principles of phase separation, therapeutic systems can establish microenvironments that enhance cell–matrix signaling pathways and improve mechanical transduction [61]. This strategic approach ensures robust tissue repair by preserving the equilibrium between matrix synthesis and degradation [83].

The dynamic interplay between ECM components and resident cells is essential for maintaining tissue homeostasis. However, traditional therapies often fall short of addressing this intricate relationship. Strategies based on phase separation effectively transcend these limitations by enabling precise modulation of matrix dynamics and cellular behavior. For instance, LLPS mechanisms can orchestrate the organization of specific matrix proteins into condensed structures, thereby facilitating localized repair [84, 85]. Similarly, engineered scaffolds that employ these principles can attract endogenous cells, directing them to areas of injury and amplifying their regenerative potential. These approaches exemplify the synergistic application of physical and biochemical principles to achieve targeted therapeutic outcomes [74, 86, 87].

Recent advancements in phase separation research have catalyzed the development of multifunctional delivery systems for bioactive substances. These innovative systems utilize phase‐separated compartments to codeliver growth factors, cytokines, or therapeutic nucleic acids, ensuring sustained release and enhancing stability within the ECM [74, 77]. By preventing premature degradation and ensuring localized activity, these systems optimize the therapeutic efficacy of the delivered molecules while minimizing off‐target effects [77, 88]. This controlled delivery approach harmonizes with natural repair processes, thereby accelerating recovery and enhancing tissue functionality.

Beyond direct repair, therapies inspired by phase separation possess significant potential for preventing chronic matrix disorders by targeting the underlying pathophysiological mechanisms. Disruptions in matrix assembly and turnover are pivotal contributors to degenerative conditions, rendering them difficult to manage with conventional methods. By reinstating phase separation–driven compartmental dynamics, these innovative strategies restore equilibrium within the ECM environment, effectively reversing pathological changes and promoting long‐term tissue health [89, 90]. This remarkable capability underscores the versatility of phase separation–based interventions in addressing a wide array of ECM‐related conditions.

The incorporation of phase separation principles into therapeutic design signifies a transformative shift in tissue repair strategies, seamlessly intertwining molecular biology, materials science, and regenerative medicine. By focusing on the dynamics of the ECM and cellular interactions, these innovative approaches transcend mere symptom management, tackling the fundamental causes of compromised healing. As research advances, the potential to translate these discoveries into clinical applications becomes increasingly promising, heralding new hope for patients grappling with complex tissue injuries and ECM disorders.

## 6. Conclusions

In recent years, the concept of phase separation has emerged as a crucial mechanism shaping the dynamics of the ECM. This review offers an extensive overview of how phase separation contributes to the organization and functionality of the ECM, presenting a novel perspective on its role in both physiological and pathological contexts.

Phase separation enables the spatial and temporal compartmentalization of ECM components, facilitating the assembly of hierarchical structures that are vital for tissue integrity and functionality. These dynamic processes are essential for preserving the mechanical properties and biochemical signaling of tissues, while their dysregulation is associated with a range of diseases, including fibrosis, tumor invasion, and osteoarthritis [39, 59, 91]. Consequently, the modulation of phase separation emerges as a promising therapeutic strategy for reinstating ECM homeostasis.

The therapeutic potential of targeting phase separation resides in its capacity to modulate the interactions and activities of proteins and other macromolecules implicated in ECM remodeling. By fine‐tuning the equilibrium between ECM synthesis and degradation, it may be feasible to rectify the aberrant matrix dynamics that typify numerous pathological conditions [78, 92]. This approach holds the promise of preserving tissue structure and function while curtailing the progression of disease.

Moreover, the principles of phase separation offer a compelling blueprint for the design of biomimetic materials and sophisticated drug delivery systems. These systems can adeptly replicate the native microenvironment of tissues, thereby enhancing the specificity and efficacy of therapeutic interventions [77, 83]. While conventional ECM‐targeting therapies, such as MMP inhibitors [18] and antifibrotic drugs [24], have shown some efficacy in modulating ECM remodeling, they often have off‐target effects, poor tissue penetration, or high toxicity. In contrast, phase separation approaches enable targeted, sustained drug release and avoid damaging healthy ECM, highlighting their unique added value [59]. In particular, phase‐separated biomaterials hold significant promise for supporting tissue regeneration by providing essential mechanical and biochemical cues critical for cellular processes [74, 88].

The integration of phase separation into the realm of ECM biology not only deepens our understanding of molecular interactions but also paves the way for novel therapeutic developments. Nonetheless, translating these insights into clinical applications necessitates a more comprehensive elucidation of the regulatory networks that orchestrate phase separation. Significant advancements in imaging technologies, computational modeling, and proteomics will be imperative in this endeavor.

In conclusion, phase separation embodies a transformative mechanism within the domain of ECM biology, harboring substantial therapeutic potential. By harnessing this process, we can devise more targeted and efficacious treatments for ECM‐related maladies, ultimately improving patient outcomes. As this field progresses, the implications of phase separation for ECM research and therapeutic interventions are not only promising but also profoundly impactful.

## Conflicts of Interest

The authors declare no conflicts of interest.

## Author Contributions

X.W., F.W., and T.Z. designed the concept. T.Z., X.W., and H.X. analyzed the data and prepared the figures. R.G., J.L., and T.Z. wrote the discussion. T.Z., F.W., and X.W. wrote the manuscript. H.X. and T.Z. conceived the project and supervised and coordinated all aspects of the work.

## Funding

This study was funded by the National Natural Science Foundation of China (Nos. 82171810 and 82473422) and the Program of Shandong Provincial Scientific and Technological Development of Traditional Chinese Medicine (M‐2023210). Fei Wu was supported by the Taishan Scholar Youth Expert Program of Shandong Province (tsqn202312349).

## Data Availability

The data that support the findings of this study are available upon request from the corresponding authors. The data are not publicly available due to privacy or ethical restrictions.
